# Module for Monitoring the Probe-Skin Contact Force in the Study of Vibration Perception on the Wrist

**DOI:** 10.3390/s21062128

**Published:** 2021-03-18

**Authors:** Dorota Czopek, Robert Barański, Jerzy Wiciak

**Affiliations:** Department of Mechanics and Vibroacoustics, Faculty of Mechanical Engineering and Robotics, AGH University of Science and Technology, Al. Mickiewicza 30, 30-059 Kraków, Poland; robert.baranski@agh.edu.pl (R.B.); jerzy.wiciak@agh.edu.pl (J.W.)

**Keywords:** force measurement, strain gauge beam, vibrotactile discrimination, smart city for blind people

## Abstract

This paper presents a module for monitoring the contact force between a probe for measuring vibration perception on the wrist and the skin. The module was designed for an original measuring stand for the automatic testing of the vibrotactile discrimination thresholds using the psychophysical adaptive method of 1 up–2 down with two or three interval forced choices (2IFC, 3IFC). Measurement methods were implemented in LabVIEW software. The inspiration for the project was the need to check the possibility of building a vibrating interface for transmitting information through vibrations delivered to the wrist via a bracelet. The test procedure on the wrist is not standardized; however, during its development, the recommendations of the Polish Norm–International Organization for Standardization PN-ISO 13091-1, 2006 were adopted. This standard contains methods for measuring vibration sensation thresholds on the fingertips for the assessment of neural dysfunction. The key to the repeatability of measurements seems to be the ability to continuously control the pressure of the measuring probe on the skin. This article compares two solutions for measuring the contact force along with an analysis of their accuracy and the impact of vibrations on the measured values. Moreover, the results of measurements of vibrotactile amplitude and frequency discrimination thresholds obtained on the ventral wrist at five frequencies (25, 32, 63, 125 and 250 Hz) are presented.

## 1. Introduction

In recent years, research related to haptic perception has been performed because of the development of techniques for augmented and virtual reality, including telepresence in military contexts and telemedicine. Haptic devices allow people to obtain a sense of touch with the virtual environment generated by a computer. By applying forces, vibrations, or motions to the user, haptic devices aid in the remote control of virtual objects and enable deeper immersion in computer-generated reality [1,2]. Many conferences are being conducted on this subject because despite the existence of considerable knowledge, there remains much to be researched in this field; to this end, the WHC World Haptics Conference is held every two years [3]. Various parameters related to the feeling of vibrations and pressure forces are tested, such as the vibrotactile perception threshold [4] and simple reaction time and response time [5]. The described research was inspired by the design of a vibrating bracelet for blind and partially sighted people, in line with the idea of a smart city for all. The system was invented to warn and inform visually impaired people about dangerous and important spots in the big city [6]. It was decided that the information must be conveyed by means of non-acoustic signals because the perception of the natural sounds of the environment is an important element in the spatial orientation of blind people. After analyzing the possibilities, based on a questionnaire and interviews not only with the target audience but also among spatial orientation teachers, a vibrating interface located on the hand or wrist was selected [7,8,9].

For the purposes of building the system interface—a vibrating bracelet—the mechanism of vibration perception on the wrist was investigated. The vibrotactile vibration absolute thresholds on the wrist were determined at eight frequencies (4, 25, 32, 63, 125, 250, 400, 500 Hz) using an P8 palesthesiometer (Emson-Mat, Skawina, Poland), according to the Polish Norm–International Organization for Standardization standard PN-ISO 13091-1, 2006 [10]). Analysis of the results of the research made it possible to limit further investigations to five frequencies: 25, 32, 63, 125, and 250 Hz. For these frequencies, further studies were conducted to investigate more details of the mechanism of perception on the wrist. It was decided to measure the vibrotactile amplitude and frequency discrimination thresholds and, for this purpose, an original measuring stand was built. The designed stand enables the measurement of the vibrotactile discrimination thresholds using the psychophysical adaptive method 1 up–2 down with two or three interval forced-choices (2IFC, 3IFC). Vibration stimuli were generated by the TMS 2004E mini modal shaker (The Modal Shop, Inc., Cincinnati, OH, USA). Automatic measurement is initiated and controlled by LabVIEW software. To ensure the reliability and repeatability of the measurement, many factors must be taken into account when designing a measurement tool [11]. Many authors have studied the factors that affect the vibrotactile thresholds on the skin [12,13]. These factors can be divided into those that are human and those that are stimulus related. Among the factors related to the examined person, the following parameters are mentioned: location on the body [14,15]; age and gender [16]; height and weight; skin temperature [17]. Among the factors related to the stimulus, the following parameters are mentioned: probe diameter; type of probe capping surface; the surround or lack of a surround around the probe; the probe-skin contact force [18,19]; stimulus frequency; the psychophysical measurement procedure [20].

In the discussed measuring system, one of the key elements influencing the obtained results is the contact force of the vibrating element (the probe transmitting the stimulus) with the wearer. As indicated earlier, its change significantly influences the perception of the stimulated person, and thus constitutes a particularly important element in the entire measurement procedure. Therefore, it is extremely important that this force is kept at a constant level for all examined cases.

## 2. Materials and Methods

### 2.1. Pilot Psychoacoustic Study

The method of constant stimuli was chosen to determine the differential thresholds in the pilot study. The high accuracy and possibility to determine not only the threshold, but also the course of the psychometric function are the main features of using such a method [21]. The study was conducted using the two-interval forced selection procedure (2IFC). The subject was presented with a series of pairs of stimuli, the first of which was the reference (standard) and had a constant amplitude, while the second was the target (test) stimulus and assumed amplitude values lower than, equal to or higher than the reference stimulus. The listener’s task was to assess the vibration intensity of the target signal and the reference signal. After the presentation of the sample, the subject determined whether the second signal given in the sequence was more intense than the first. A response that the signals were the same was not allowed. The respondent’s responses were interpreted as follows: (yes) positive answer—the vibration intensity of the target signal is greater than the reference signal intensity; (no) negative answer—the vibration intensity of the target signal is less than the reference signal intensity.

The study of differential thresholds was performed for the five selected frequencies (25, 31.5, 63, 125, 250 Hz) selected on the basis of previous research [6], but the pilot series was limited to two frequencies (31.5 and 125 Hz). The selected frequencies allowed us to study the differential thresholds of fast-adapting Meissner mechanoreceptors (FAI—25, 31.5 and 63 Hz) and fast-adapting Pacinian mechanoreceptors (FAII—125 and 250 Hz) [22]. Pilot studies were performed for the value of the standard stimulus vibration acceleration level 10 dB higher than the absolute vibrotactile perception threshold (VPT) for a given frequency. The values of the vibration acceleration of the comparative stimuli are presented in Table 1.

**Figure 1:**
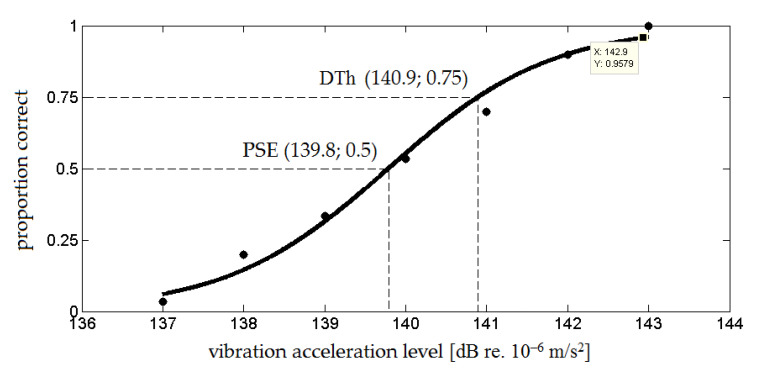
Example course of a psychometric function with marked the point of subjective equality (PSE) and the difference threshold (DTh) (logistic function was adjusted to the data).

Each pair with different amplitude levels of the acceleration of the compared stimuli was presented a total of thirty times. The level of the amplitude of the target stimulus was randomly changed. In order to interpret the results, the course of the psychometric function was fitted based on the measurement data (Figure 1). The points through which the function passes were obtained by calculating the percentage of positive responses for each value of the target stimuli. The course of the psychometric function was obtained by adjusting a sigmoid function to the obtained points. The logistic function was chosen a priori based on a theory of the “true” internal shape of the psychometric function and experiment conditions [23].

The values of the vibration acceleration amplitude level for the point of subjective equality (PSE) and the difference threshold (DTh) are the arguments for which the psychometric function assumes the values of 50% and 75%. The Just Noticeable Difference (JND) in the vibration acceleration amplitude was calculated as the difference between the difference threshold value and the point of subjective equality value [18,23].

**Figure 2:**
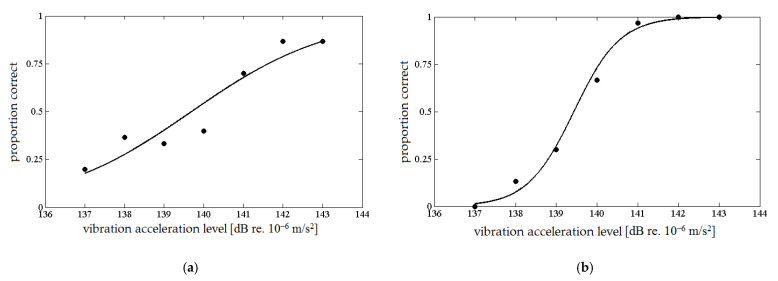
The course of the psychometric function—example results obtained in the pilot tests: (**a**) for subject that did not achieve 100% correct indications for the stimulus pairs with the largest amplitude difference and (**b**) for subject that almost unmistakably indicated a more intense stimulus even for pairs of stimuli with the smallest difference in amplitudes.

After conducting the pilot tests, it was observed that the vibration amplitude on the wrist is characterized by a large variation. It was found that it is difficult to maintain and control a constant level of pressure of the probe on the skin. Additionally, a large problem in using the method of constant stimuli is the selection of the amplitudes of the compared stimuli. The values of the stimulus amplitudes were changed several times; however, satisfactory results were not achieved. If for one person the differences between the amplitudes of the stimuli allowed the psychometric curve to be drawn correctly, there were problems for another. Some subjects did not achieve 100% correct indications for the stimulus pairs with the largest amplitude difference (Figure 2a), while others unmistakably indicated a more intense stimulus even for pairs of stimuli with the smallest difference in amplitudes (Figure 2b). 

The reason for the large discrepancy in the results for individual subjects obtained in the pilot study may be twofold. An important factor was probably the inaccuracy of the measurement of the probe-skin contact force, and thus insufficient accuracy in the control of the level of this force during the test. For this reason, the test stand, in particular the method of controlling the pressure of the measuring probe on the skin, also required modification. In addition, measurements of the vibrotactile amplitude and frequency discrimination thresholds were performed using the adaptive 1 up–2 down method.

### 2.2. Main Psychoacoustic Study

The adaptive 1 up–2 down method was chosen to determine the differential thresholds in the main study. As a stimulus, a one-second vibrating sinusoidal measurement signal was used with frequencies of 25, 32, 63, 125, and 250 Hz. In the amplitude study, various intensities within the range of 100–150 dB ref 10^−6^ m/s^2^ were used. In the frequency study, frequencies varied in the range of 100–150% of the reference frequency. The stimulus was applied to the skin by means of a stimulation probe shaped like a flat cylinder with a diameter of 5 mm. The test was performed with a probe without a surrounding with a controlled force of contact with the skin in the range of 0.1–0.2 N. 

**Figure 3:**
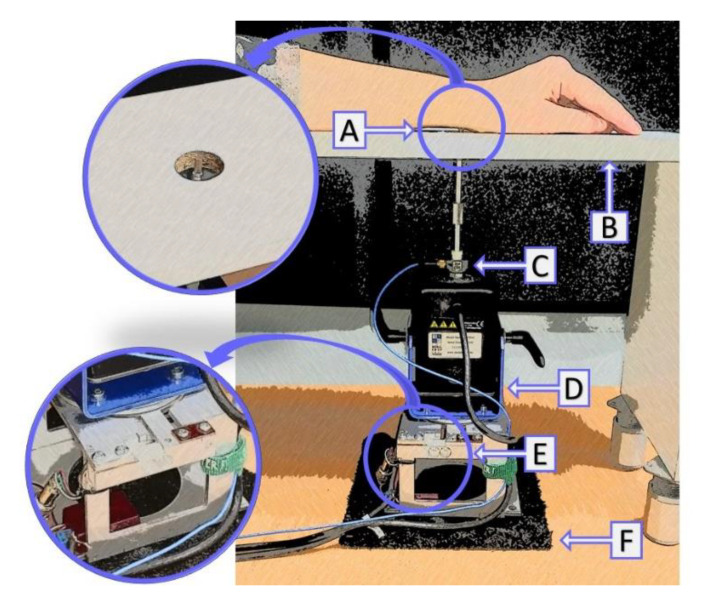
The illustrative sketch of the measuring stand for testing the differential vibration thresholds on the wrist: (**A**)—probe, (**B**)—support of the subject’s forearm, (**C**)—accelerometer PCB M354C03, (**D**)—modal shaker TMS 2004E, (**E**)—the probe-skin contact force monitoring module, (**F**)—vibration insulation.

The measuring stand designed for the adaptive 1 up–2 down study of vibrotactile amplitude and frequency discrimination thresholds on the wrist is shown in Figure 3. The illustrative sketch of the measuring stand shows elements of the stand: A—probe, B—support of the subject’s forearm, C—accelerometer PCB M354C03, D—modal shaker TMS 2004E, E—the probe-skin contact force monitoring module, F—vibration insulation. The measuring procedure was controlled by the computer with LabVIEW software with the 4-channel voltage output module NI 9263 (National Instrument) and the 4-channel input module NI 9234. NI 9263 was used to send the vibration stimuli to the mini modal shaker TMS 2004E (The Modal Shop) through the Apart MB-150 amplifier. NI 9234 was used to control the measurement conditions. Additionally, the masking signal (pink noise) was sent through the computer sound card to the Beyerdynamic DT 770 pro headphones to exclude the use of audio cues by the subjects.

For the presentation of the stimuli, the two-interval forced choice procedure (2IFC) for amplitude discrimination and the two-interval forced choice procedure (3IFC) for frequency discrimination were used. In the trial, the reference and target stimuli were presented in a random order. The listener’s task was to assess which of the signals was stronger (amplitude) or different (frequency). The difference in the vibration acceleration level and the frequency of the reference signal and the target signal in the first step were determined in such a way that all subjects clearly felt which signal was stronger or different and gave the correct answer. In the main phase of the procedure, a reduction in the difference between the stimuli could only take place after two consistent, correct answers. However, after each incorrect answer, or a sequence of correct and incorrect answers, the difference between the stimuli in the next trial increased. The vibrotactile discrimination threshold for each tested amplitude frequency was calculated as the arithmetic mean of the stimulus value from all reversals, omitting the first two. The study was completed after eight reversals.

### 2.3. Contact Force Measurements

The method with electrofusion elements working in a bridge system is most often used to determine the force (commonly known as strain gauge bridges). The principle of the operation of strain gauge bridges is based on measuring changes in the resistance of electrofusion elements as a result of changing deformations and displacements. 

When the strain gauge is properly mounted (usually it is a glued joint), its deformation is identical to the deformation of the sample on which it is glued. Therefore, with strain, there is a proportional change in resistance.

**Figure 4:**
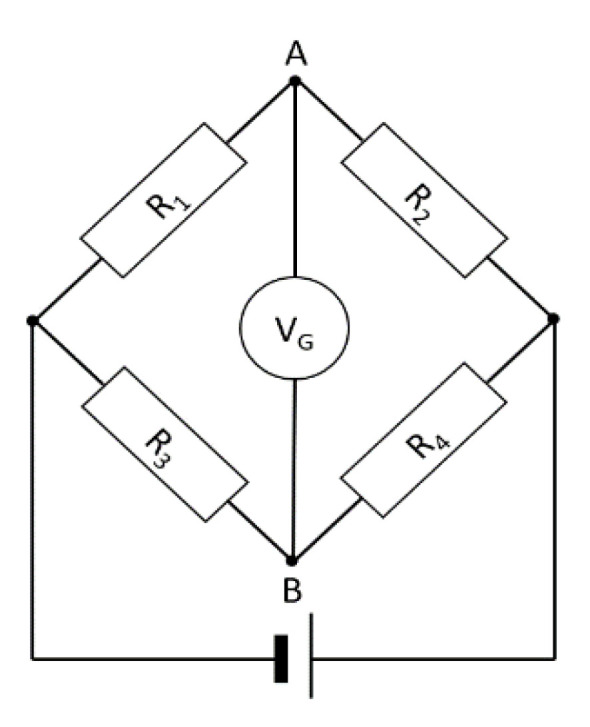
Schematic diagram of the Wheatstone bridge circuit.

Due to the very low deformation values, the changes in resistance are also small. Therefore, measuring them directly is ineffective. In connection with the above, it is normal to operate a strain gauge in a bridge system. The Wheatstone bridge is most often used as the input circuit (Figure 4).

On the presented circuit, one strain gauge is glued to identify deformations (R1), while another (R2) does not respond to deformations (compensate for the influence of temperature). Assuming all resistances are equal (R = R_1_ = R_2_ = R_3_ = R_4_), the bridge is balanced, and the potential difference between terminals A and B (U_AB_) is 0 V. In the event of stress, the resistance R_1_ will change so that the equilibrium of the bridge will be disturbed. In practical applications, the voltage U_AB_ is always different from zero, while the occurrence of stress causes a change in this tension, which is linearly dependent on the stress (for small elastic strains). The dependence of the change in this voltage can be expressed as:

(1) $${\Delta U}_{\mathrm{AB}}=\frac{U}{4}\frac{{\Delta R}_{\varepsilon }}{R},$$

where:

∆U_AB_—voltage change due to stress (V);

∆R_ε_—resistance change due to stress (Ohm);

R_1_, R_2_, R_3_, R_4_—resistances in the branches of the bridge (Ohm).

Very often, the practical use of strain gauges working in a bridge system comes down to the use of so-called strain gauge beams. In this study, the NA27 beam was used, which is characterized by its small dimensions (12.7 × 12.7 × 80 mm). The range of available loads is from 0.6 kg to 6 kg.

As previously mentioned, signal amplifiers are used to register the very small changes occurring in the resistance of the resistance element under the influence of the stress. Amplifiers make it possible to amplify the U_AB_ voltage so many times that its change is easily detectable by an analogue-to-digital converter.

In addition to the measurement cards, which are dedicated to the measurement of stresses and loads (NI-9237, C Series Strain/Bridge Input Module) with the use of strain gauges working in a bridge system, there is the option to use standard measurement cards, which, together with the conditioning system, can be an excellent alternative. Another solution is to use a ready-made module based on the HX711 chip.

#### 2.3.1. Analog Amplifier—AnAmp

In the approach based on the use of an amplifying circuit, practice shows that low-pass filtration should be applied on the input signal. In the presented application, the changes in forces can be classified as slowly changing waveforms with expected frequencies not greater than a few Hz. In this case, filtration allows for the elimination of artefacts resulting from external disturbances (e.g., electromagnetic artefacts).

**Figure 5:**
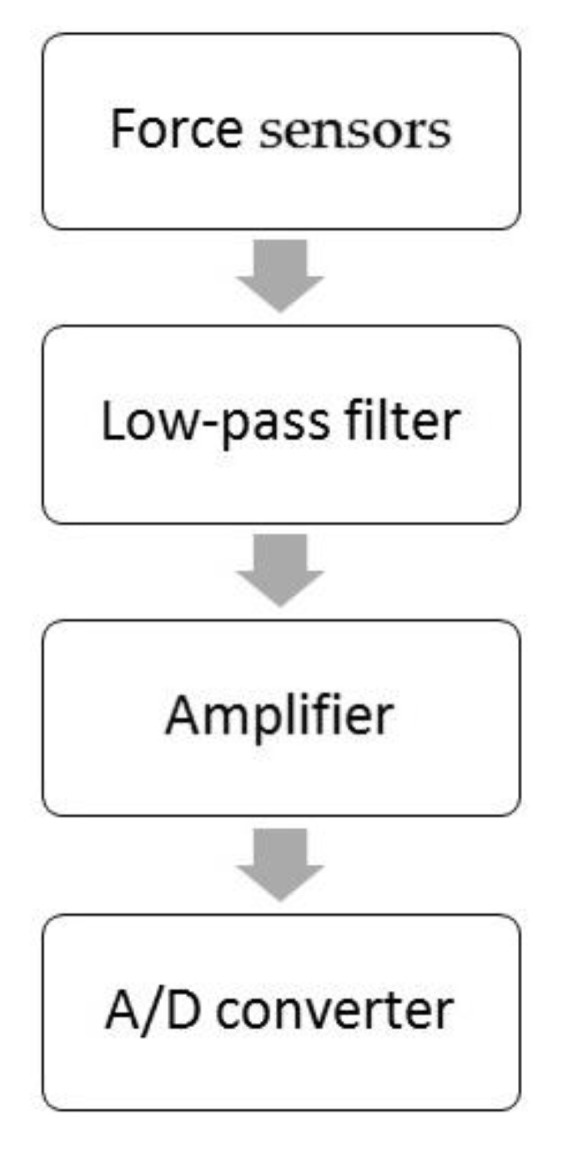
Signal conditioning diagram.

**Figure 6:**
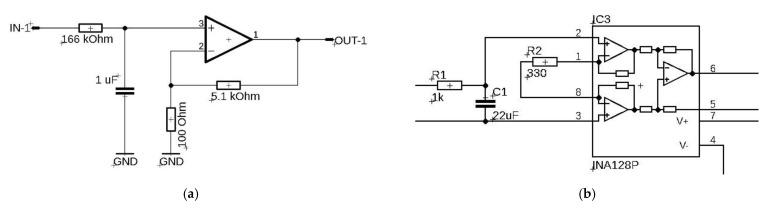
Low-pass filter and a general diagram of an operational amplifier: (**a**) based on TL084; (**b**) based on INA128P.

The approach presented in Figure 5 has been successfully used to build an active rehabilitation platform (Weight-Shifting Exercises with Compensatory Forces Monitoring) [24]. In this case, the whole approach was simplified as much as was possible by using a passive filter (first order) and a standard TL084 operational amplifier (Figure 6a). For the values selected in this way, the cut-off frequency of the low-pass filter was equal to 1 Hz and the gain at the level of G = 51 (Figure 6).

The filtration and measurement system can also take a more complex form and use a precise measuring amplifier characterized by, among other features, high CMRR >100 dB (common mode rejection ratio). In the case presented above (Figure 6b), the selected parameters of passive filtration give the cut-off frequency at the level of 7.6 Hz, while the gain is achieved by the resistor R2 and, in our case, it was 150.

Solutions based on our own gain stage require the use of an analogue-to-digital converter. In the case of our research, the NI USB 6212 BNC measurement card was used, with a 16-bit converter with a maximum sampling frequency of 400 kHz. Preliminary studies have shown that the changes in the signal coming from the analog amplifier are at least two orders of magnitude greater than the accuracy of the 16 bit converter used at the voltage range of ±10 V. As mentioned before, due to the expected rate of force signal changes, a sampling frequency of 20 Hz would be sufficient.

It should be emphasized that in the case of measurements with a strain gauge beam, it is extremely important to ensure a stable power supply to the strain gauge bridge system. In the case of the HX711 chip, the chip itself takes care of it. However, if one uses their own analogue amplifier, they need to take care of it themselves. The solution may be to use the popular LM78L05 voltage stabilizer with an output voltage of 5 V. For measurements lasting several dozen minutes with an average voltage of 5.02 V, the standard deviation was 1 mV (0.2% of the supply voltage), and the percentage coefficient of variation was only 0.002%. It was concluded that such small changes in the supply voltage should not have a noticeable effect on the results.

#### 2.3.2. Conditioning Module—HX711

Currently, in the case of the construction of systems for measuring forces with the use of strain gauge beams, the HX711 electronic system has become very popular [25]. This system includes the previously mentioned modules without low-pass filtration. It contains a programmable amplification circuit (low-noise programmable gain amplifier (PGA)). The input channel can be programmed with a gain of 128 or 64, corresponding to a full-scale differential input voltage of ±20 mV or ±40 mV. The system is equipped with a 24-bit analogue-to-digital converter. It should be noted that due to the disturbances of the measured signal, and due to the influence of electromagnetic fields and the power stability, etc., it is impossible to obtain the theoretical resolution (resulting from the resolution of the converter). However, in combination with a standard strain gauge, the obtained measurement resolutions are sufficient for most applications.

**Figure 7:**
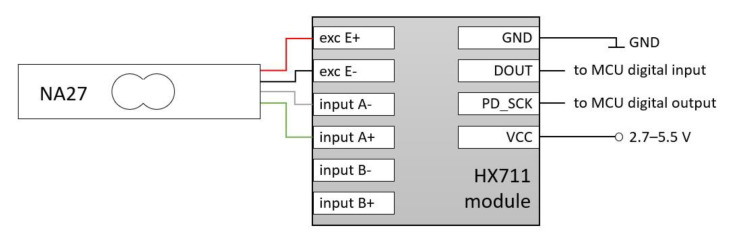
Connection diagram of strain gauge beam NA27 and HX711 module (exc—strain gauge beam excitation; input A—analog input; GND—ground, DOUT—Serial Data Output; PD_SCK—Power Down and Serial Clock Input; MCU—microcontroller unit, VCC—Voltage Common Collector).

Figure 7 shows a block diagram of an example connection of a strain gauge bridge (Load cel). Although communication with the HX711 chip is digital, the designers do not use registers to change the system parameter settings but use the available pins instead—there is no programming needed for the internal registers and all controls to the HX711 are through the pins. The serial communication protocol described in the HX711 datasheet is used for communication [25]. When output data are not ready for retrieval, the digital output pin DOUT (Serial Data Output) is high. Serial clock input PD_SCK (Power Down and Serial Clock Input) should be low. When the DOUT becomes too low, it indicates that data are ready for retrieval. By applying 25~27 positive clock pulses at the PD_SCK pin, data are shifted out from the DOUT output pin. Each PD_SCK pulse shifts out one bit, starting with the most significant bit first, until all 24 bits are shifted out. The 25th pulse at the PD_SCK input will pull the DOUT pin back to high (Figure 7). Its implementation in the Arduino Integrated Development Environment (IDE) may look like this:


long int mea_HX711(int pomH_SCK, int pomH_DOUT) {
  long int x = 0;
  digitalWrite(pomH_SCK, LOW);              //SCK setting to LOW
  while (digitalRead(pomH_DOUT) != LOW)      // waiting for the state HIGH line Data
  {}
  for (int i = 0; i < 24; i++)             // reading 24-bit data from HX711
  {
    digitalWrite(pomH_SCK, HIGH);          // generating a HIGH pulse of the clock
    digitalWrite(pomH_SCK, LOW);           // generating a LOW pulse of the clock
    bitWrite(x, 0, digitalRead(pomH_DOUT));     // reading data bit
    x = x << 1;
  }
  digitalWrite(pomH_SCK, HIGH);
  digitalWrite(pomH_SCK, LOW);
  return x;
  }
		  

In the above code, pomH_SCK is defined as digital output and pomH_DOUT is defined as digital input. In our case, an ATmega328P microcontroller was used as a management system, as is installed as standard in Arduino UNO modules. It is worth emphasizing that the values returned by HX711 should be scaled to be useful. In the case of force measurements using a strain gauge beam, this scaling is most often performed by calibrating the system by measuring two known masses and then determining the coefficients of the function determining the conversion of data from HX711 to mass values.

### 2.4. Contact Force Measurements—Accuracy

The solutions presented above are part of a system dedicated to controlling the forces of contact of the vibrating element with the human body. Special attention was paid to testing reliability in the development of a new measurement system.

The quality of the measurement system is the accuracy of the measurement and the variance of the results obtained when measuring the same quantity. The variance of the results is influenced by the environment (e.g., temperature, humidity, pressure) as well as by the person performing the measurements. The basic errors of the measurement system influencing the variance of the results are accuracy, repeatability, reproducibility, stability and linearity [26,27,28].

### 2.5. The Vibrotactile Perception Threshold—Psychophisical Procedure

The discrimination of changes in the vibration amplitude or frequency is based on the ability of the sense of feeling to perceive changes in vibration amplitude or frequency over time. In other words, this ability allows a person to notice the difference in amplitude or frequency of two vibration signals [29].

The measurements of the vibrotactile amplitude and frequency discrimination thresholds were performed using the adaptive 1 up–2 down method [23,30]. The vibrotactile amplitude discrimination thresholds were determined using the two-interval forced-choice (2IFC) procedure, and vibrotactile frequency discrimination thresholds were determined using the three-interval forced-choice (3IFC). The choice of the procedure was made due to the difference in the perception of changes in the amplitude and frequency of the vibrational stimulus. Changes in the vibration signal amplitude are well defined and well recognized. By contrast, changes in the vibration signal frequency are poorly recognized by the human sensory system. Therefore, a 3IFC procedure was used in which the subject indicates a stimulus which is different from the other two but does not have to be able to define the difference. The schematic diagram of the stand and the details of the measurement procedure with regard to the methodology of the psychophysical research have been described in separate articles [31,32,33].

## 3. Results

### 3.1. The Contact Force Measurements

Measurements were performed to determine the metrological measurement capabilities of the two previously presented solutions. At the outset, it should be emphasized that the target tests were performed using a probe with a controlled contact force with the skin in the range of 0.1–0.2 N (the standard allows 0.06–0.24 N [10]). The measurements were made by loading the system with the following weights: 1.0 g (0.00981 N), 2.0 g (0.0196 N), 5.0 g (0.049 N), and 10.0 g (0.0981 N). The weights were previously verified using a jewelry scale. Load values with an order of magnitude lower than the target load were deliberately selected.

Twenty measurement series were completed. Each series contained ten repetitions of the measurement; between each repetition, the system load was temporarily and randomly changed (e.g., by removing the load and replacing it several seconds later). Each repetition of the measurement took ten seconds. Every second value is the average value of the signal recorded at 10 Hz.

**Figure 8:**
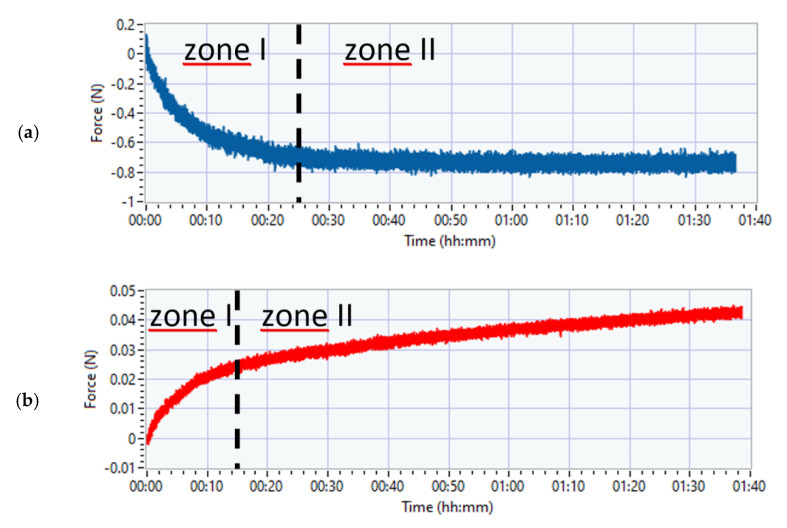
The nature of the signal changes from when the system is powered up: (**a**) AnAmp; (**b**) HX711.

In the case of measurements with the above circuits (analogue amplifier and HX711), the characteristic of changes of the signal from the unloaded measurement circuit is not obvious. Figure 8a,b shows example waveforms from the moment the device is powered on, without additional system loads. The measurements were performed several times and each time the results were consistent. Analysis shows two compartments (zone I and zone II in Figure 8).

In zone I, the signal changes rapidly. Zone II is an almost rectilinear dependence of the signal value change over time. The figures also show the range of changes, which in the case of the analogue amplifier is close to 0.8 N in comparison to 0.04 N for the HX711. A constant component created in this way is easy to correct. The data show that the differences between the systems are significant.

**Figure 9:**
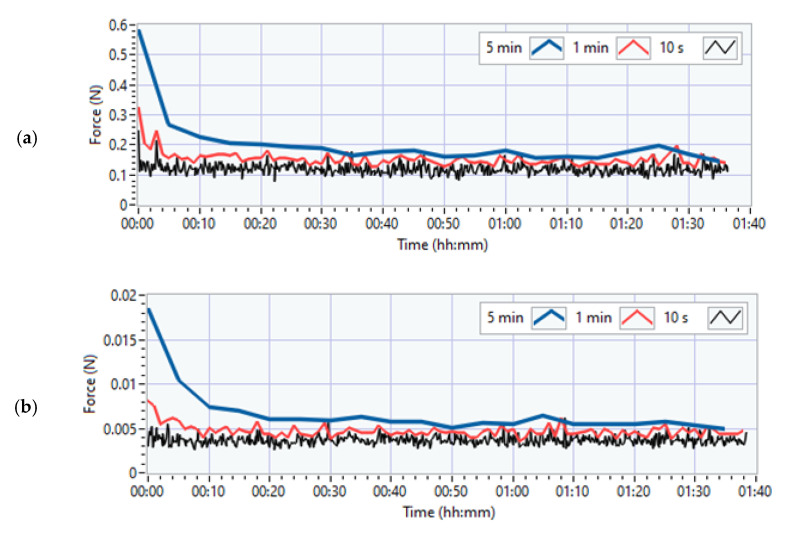
Changes in max-min in time intervals: 5 min (blue), 1 min (red), 10 s (black): (**a**) AnAmp; (**b**) HX711.

In Figure 9, in addition to the changes in the constant component of the signal over time, there are also visible disturbances of the measured value, taking the form of wideband noise. Importantly, it can be assumed that the occurring disturbances remain unchanged throughout the research period, as evidenced by the chart below showing the values of the maximum differences in the signal (max-min) for 10 s intervals (black). Differences for different time intervals (10 s, 1 min, 5 min) are also presented. For longer periods of time (1 min, 5 min), zone I becomes noticeable, i.e., the phase of dynamic change of value that occurs immediately after switching on the power to the system.

It can also be read from the above graphs that the interval of the recorded signal in the case of the time window of 5 min (in the case of no direct current component compensation), for the AnAmp solution, is in the order of 0.2 N, while in the case of HX711, it is close to 0.007 N. It is worth emphasizing that the tests of vibrotactile discrimination thresholds (0.1–0.2 N) for one acceleration value do not last longer than 5 min. However, the above graph shows that the error resulting from the lack of compensation for 5 min in the case of the AnAmp system may lead to erroneous conclusions because the level of the measured signal fluctuations will hinder the force control. In the case of the HX711 system, this problem does not exist because the signal fluctuations are over an order of magnitude smaller than the measured value.

In order to determine the accuracy of the measurement as a standard expressed as the difference between the average value of measurements and the actual value of the measured quantity, several measurement series were made for the above-mentioned loads.

The determined accuracy (bias) for all registered series and repetition of measurements for the AnAmp system did not exceed 0.105 N. In the case of the HX711 system, the determined bias did not reach the maximum value of 0.013 N. Thus, in the case of the AnAmp system, the accuracy of the measurement was again close to the measured value (0.1–0.2 N), while in the case of HX711, it was an order of magnitude lower.

### 3.2. The Vibrotactile Amplitude and Frequency Discrimination Threshold Measurements

**Figure 10:**
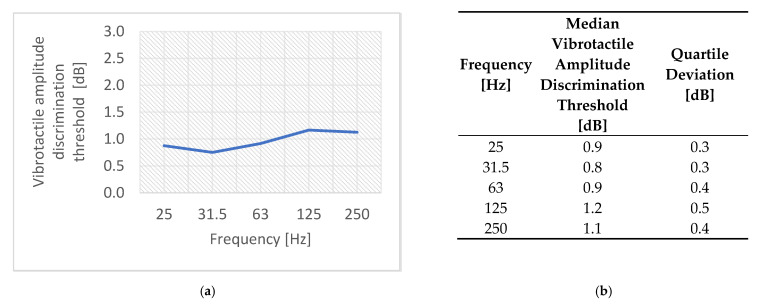
The vibrotactile amplitude discrimination thresholds: (**a**) median value and (**b**) tabular summary of median and quartile deviation values.

**Figure 11:**
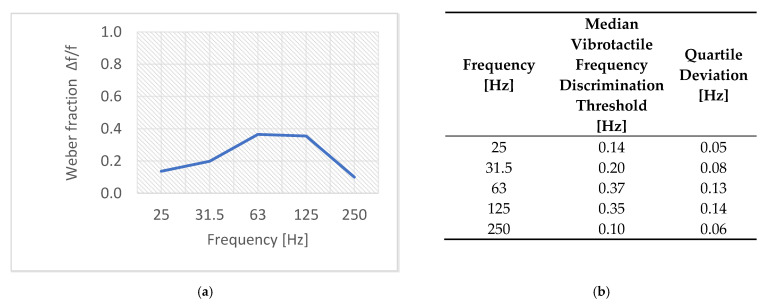
The vibrotactile amplitude discrimination thresholds shown as Weber fraction Δf/f thresholds: (**a**) median value and (**b**) a tabular summary of median and quartile deviation values.

The research group consisted of thirty people aged from eighteen to twenty-eight years: fifteen women and fifteen men. The median value of the vibration amplitude discrimination threshold is shown in Figure 10. The median value of the vibration frequency discrimination threshold is shown in Figure 11 using the Weber fraction because comparing threshold values in Hz is difficult. The Weber fraction is equal to the ratio of the just noticeable difference (discrimination threshold value) to the frequency of a stimulus Δf/f [18,23].

There were slight differences in the threshold values in the range from 0.8 to 1.2 dB. Therefore, the results were statistically tested [31] and it was concluded that there are no statistically significant differences between the most tested frequency pairs. The vibrotactile amplitude discrimination threshold is statistically significantly lower only for 31.5 Hz versus 125 Hz. This could be explained by the mechanism of sensation and the fact that vibrations at 31.5 Hz are perceived by fast-adapting Meissner mechanoreceptors, while vibrations at 125 Hz are perceived by other fast-adapting Pacinian mechanoreceptors. After analyzing and discussing the obtained results, it can be concluded that the just-noticeable difference of vibration amplitude on the wrist is around 1 dB.

The results shown in Figure 11 show that the value of the Weber fraction Δf/f increases with the frequency from 25 Hz up to 125 Hz, and decreases for the frequencies from 125 Hz to 250 Hz. After analyzing the obtained results (confirmed by statistical tests [32]), it can be concluded that there are some statistically significant differences between the vibrotactile frequency discrimination thresholds for the tested frequency pairs. There are statistically significantly lower thresholds for 25, 31.5 and 250 Hz than for 63 and 125 Hz.

## 4. Discussion and Conclusions

The influence of probe vibrations on contact force at the moment when subjects were exposed to vibrations was measured and analyzed. The impact of vibration was investigated only for this HX711 system because it was the system which demonstrated the best measurement properties.

**Figure 12:**
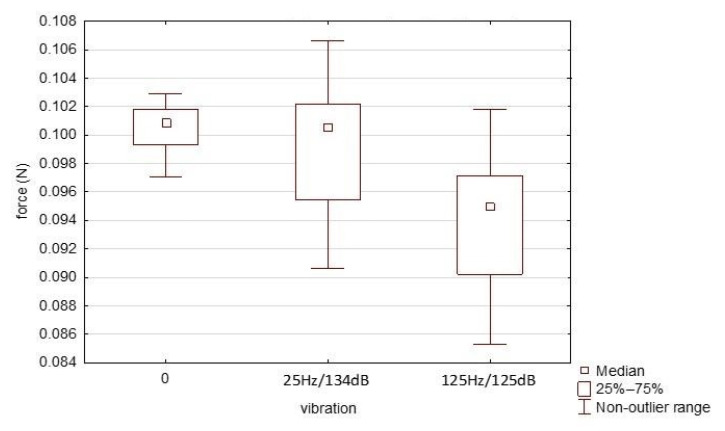
Distribution of forces in the presence of vibrations.

Measurements were made for a target load close to 0.1 N and equal to 10.0 g (0.0981 N). Two of the vibration levels were selected—25 Hz and 134 dB (5.01 m/s^2^) and 125 Hz and 125 dB (1.78 m/s^2^). The results for the three levels are presented in Figure 12 (the results were compared with the absence of vibrations—0.0 m/s^2^).

In the case of statistical analyses, it was decided to use a one-way ANOVA for intergroup comparisons. This required the studied variables to have normal distributions. The performed W Shapiro–Wilk test gave grounds to reject the hypothesis that all three distributions are normal. Therefore, the median test was performed. This statistic, for hypothesis H0, will test whether all studied samples come from populations with identical medians. The results obtained indicated that H0 should be rejected. 

To sum up, the influence of the vibration level on the obtained force measurement results is significant. It should be noted, however, that the results relating to accuracy presented earlier are based on measurement series that also included series determined during the occurrence of vibrations. Thus, despite the fact that their influence on the measured values has been demonstrated, in the opinion of the authors, they do not affect the use of the presented system for testing the vibration sensing thresholds.

The psychophysical tests performed on the designed measuring stand were used to deepen understanding of the mechanisms of the human perception of vibrations on the wrist. Two experiments investigating the perception of changes in vibration parameters of vibrations on the wrist were conducted, focusing on vibrotactile amplitude and frequency discrimination thresholds. These thresholds were determined for five standard frequencies: 25, 32, 63, 125 and 250 Hz. Based on the analysis of the results of the described experiments, a vibrating interface to the system was designed to inform visually impaired people about dangerous and important locations [6].

Devices for the measurement of human vibrotactile thresholds are not formalized. In the literature, one can find publications in which the results of research on determining the vibrotactile thresholds are presented, but both the measurement procedures and used devices are individually selected by scientists each time. Examples of vibrotactile detection methods are shown in the publications [4,16,34]. In the indicated publications, the measurement procedure and, in particular, the technical solutions used have not been described in detail, and the application of similar solutions could be difficult. Additionally, there are no commercial solutions that enable place of application of measurements such as fingers, wrist, forearm, back, feet, but also the face in one device. Therefore, there is a need to indicate a generally available solution that, after slight modifications, could also be used by other research institutions. In our opinion, such a solution is the use of generally available measuring modules such as strain gauge beams or the HX711 module—available and low cost. The goal of the described solution is to indicate the possibility of using modular solutions available on the market that can be easily adapted to your needs.

Summarizing the above-presented analyses of the structure of the system, as well as its measurement capabilities in the context of measuring forces in the expected range of 0.1–0.2 N, it is clearly indicated that the use of the HX711 system is an approach that would allow for much greater accuracy of the measured quantities than the use of the AnAmp system. In the case of using an analog amplifier (AnAmp), one should take into account the change in the recorded signal resulting from random interference in the signal transmission path between the elements of the amplifier circuit and the noise of the amplifier circuit. The power supply stability of the strain gauge is also critical. Its fluctuations directly affect the obtained results. Therefore, if you decide to use an analog amplifier circuit, it is necessary to ensure a stable voltage supplying the measuring system.

In the case of the HX711 module, due to the fact that all the electronics are contained in one integrated circuit, it can be assumed that the interference in the signal transmission path between individual components of the circuit is kept to a minimum. In addition, the HX711 module has a built-in precise on-chip power supply regulator, which reduces errors resulting from the instability of the power supply of the strain gauge bridge system to a minimum. As a result, the HX711 system allows us to obtain five times smaller data scatter values and accuracy than the measured quantity. This allows us to assume that the credibility of the obtained results would allow the assumed test criteria to be maintained.

## Figures and Tables

**Table 1:** The parameters of reference and target stimuli used in the pilot experiment investigating the vibrotactile amplitude discrimination thresholds.

No.	Signal Frequency	Vibration Acceleration Level of Reference Signal [dB re. 10^−6^ m/s^2^]	Vibration Acceleration Level of Target Signal [dB re. 10^−6^ m/s^2^]
1	31.5	140	137, 138, 139, 140, 141, 142, 143
2	125		

## Data Availability

Not applicable.
